# Entrepreneurial tendency across the adult lifespan

**DOI:** 10.1371/journal.pone.0262856

**Published:** 2022-02-02

**Authors:** Yaron Zelekha, Gitit Kavé

**Affiliations:** 1 Ono Academic College, Kiryat Ono, Israel; 2 The Open University, Ra’anana, Israel; Washington University, St. Louis, UNITED STATES

## Abstract

This study examines whether age associates with entrepreneurship tendencies across the lifespan, after taking into account aspects of personality that affect entrepreneurship. Participants (N = 963) aged 18–81, including 200 actual entrepreneurs, completed questionnaires about entrepreneurship tendency, personality traits, and attachment orientations. Results show that age is associated with a reduced tendency to engage in entrepreneurial activity. However, this decline is quite limited, it weakens with age, and is absent after age 50. In addition, the negative association of age with entrepreneurial tendency is smaller in participants with above-median entrepreneurship tendency scores relative to those with below-median scores, and it disappears in actual entrepreneurs. Furthermore, most of the traits that have been previously associated with entrepreneurial tendencies, especially Openness to Experience and Extraversion, remain unchanged with age, accounting for the stability of entrepreneurial tendency over time. The results have implications for policy makers who wish to encourage older adults to engage in entrepreneurial activity.

## Introduction

The role of older adults in the labor market has attracted a growing interest, due to changes in population structure in many countries. Increased longevity affects the percentage of individuals who belong in the working force, as well as the time left for older adults to live (and work) after official retirement. In order to face the socioeconomic challenges associated with population aging, policy makers and scholars have suggested that there should be greater emphasis on entrepreneurship among older adults [1–4].

Despite the importance of entrepreneurship in older age, global figures show that most entrepreneurs are young [5–9]. There is no consensus as to the exact age at which an entrepreneur is considered old, but the level of entrepreneurial activity after age 50 is about half its level among adults aged 20–49 [10]. Moreover, countries with a higher average population age have lower rates of new business formation or entrepreneurial activity, and this is true in both developed and developing countries [11–13].

Theoretically, there can be three possible reasons for the negative correlations between age and entrepreneurship: external societal barriers to entrepreneurial activity (e.g., discriminatory practices and social exclusion); internal barriers to entrepreneurial execution which affect the entrepreneurial execution skills (e.g., human or social capital); and a declining tendency to engage in entrepreneurship (e.g., entrepreneurial motivation, entrepreneurial vision or entrepreneurial awareness). The main questions of the current study are whether the tendency to engage in entrepreneurship is associated with age, and whether this association is stable after taking into account personality traits that affect entrepreneurship.

## Background

The entrepreneurship literature describes three major stages or processes of entrepreneurial development that involve (1) discovery or identification of opportunity [14–17]; (2) validation and development of this opportunity [18–24]; and the (3) execution stage in which a new business is established [14, 16, 24].

The literature on entrepreneurial tendency in old age has traditionally focused on entrepreneurial motivation, with an emphasis on the Opportunity Cost of Time [OCT model, 8]. According to the OCT model, older people are less willing to invest their effort in entrepreneurial activity because they have less time to make a return, or because they view the time left for them to live as insufficient. That is, their time is more costly. This explanation is closely related to evidence that shows that older people tend to demonstrate an increased risk aversion in decision making, especially in decisions that concern investments [25, 26]. If older adults view their time as limited, they prefer not to invest money in endeavors with long-term returns.

Yet, there are several reasons to expect greater entrepreneurial activity in older age that may balance the cost of time effect.

First, retirement is associated with a decrease in income [4], as well as with difficulties in participation in the traditional labor [1, 27]. Older employees are seen as less likely to change, less likely to keep up with technology, or less likely to succeed in positions that require creativity and innovation, and such stereotypes result in discrimination against older employees [28]. These aspects should provide an incentive to engage in entrepreneurial activity. In fact, [1] argue that in some cases, "starting up a business may be the only alternative for mature individuals wishing to resume economic activity". Indeed, necessity has been shown to encourage entrepreneurial activity, and individuals who cannot find jobs in traditional labor markets will tend to demonstrate higher rates of entrepreneurship, as is the case with immigrants [29].

Second, experience [30], as well as financial and social capital [31, 32] increase with age, and could thus make entrepreneurial activities more feasible, especially for people with high entrepreneurial tendencies who can use these means. Therefore, it is essential to examine people with high entrepreneurial tendency in compared to people with low entrepreneurial tendency.

As people age, human capital, and particularly business experience, become more valuable. Experience helps entrepreneurs identify and exercise business opportunities [30]. We note that although the literature emphasizes that tendency and success in entrepreneurship depend mostly on domain-specific human capital [32–34], it is unclear whether older people have domain-specific human capital which is necessary for entrepreneurship. Physical capital is important for financing business initiatives, especially since ageism might prevent older adults from obtaining credit. Social capital is even more important since social ties can facilitate more optimal partners, investors, customers, suppliers, and even employees, who are all essential for entrepreneurial success [31, 32, 35]. In principle, these increases in human, physical, and social capital could improve entrepreneurial activity and offset some of the effects of the increase in cost on entrepreneurial motivation. Thus, internal barriers in the form of less human, social, and financial capital may actually decrease with age.

Furthermore, the entrepreneurship literature stresses the importance of non-monetary rewards that accompany entrepreneurship [36]. Older adults may wish to continue working not only because of financial reasons but also because they prefer to remain active and because they view work as a way to teach, train, and share skills with younger employees [37]. Therefore, motivations for starting new organizations in older age are varied, and include meeting a social challenge or helping others, as well as striving for greater autonomy and for overall well-being in later life [38].

While the cost of time negatively affects entrepreneurial motivation in old age, necessity, resources, and rewards may have positive effects on entrepreneurial motivation in old age. These two opposing forces may not start to affect people simultaneously, and therefore the combined effect can either accelerate or attenuate over time.

Thus, we hypothesize that:

*H1a*: *The association between age and entrepreneurial tendency scores will be negative although not stable over time*.

In addition, the literature presents neither theoretical justifications nor empirical findings that support an inevitable and universal decline in working motivation along the lifespan [39]. In contrast, the literature on the effects of age on personality traits has generally emphasized the stability of personality traits over the adult years [40, 41]. The estimated effect size for the Big Five construct as a set explains 13% of the variance in entrepreneurial intention (Zhao et al., 2010; Zhao and Seibert, 2006). Indeed, it has been found that entrepreneurial activity increases with age for people who prefer to be self-employed and decreases with age for people who prefer to be hired by others [42, 43]. These findings suggest that we should consider personality traits when examining entrepreneurial activity in older age.

Thus, we hypothesize that:

*H1b*: *The contribution of personality aspects to the estimation of entrepreneurial tendency scores will be significantly larger than the contribution of age*, *accounting for most of the variance in entrepreneurial tendency scores*.

### Entrepreneurship and personality

Previous research has identified several personality traits that associate with entrepreneurship [35, 42–47]. Meta-analyses suggested that entrepreneurs differ from managers in terms of four personality dimensions. Entrepreneurs score higher on Conscientiousness and Openness to Experience and lower on Neuroticism and Agreeableness. No difference has been found for Extraversion [42, 43]. However, these meta-analyses have compared entrepreneurs and managers, without looking at the general population. In fact, it has been shown that managers score higher than the general population on Extraversion [48]. It is thus possible that while entrepreneurs do not differ in Extraversion from managers, they, like managers, show higher Extraversion scores when compared to the general population. Indeed, the characteristics of Extraversion (assertiveness, sociability, the tendency to seek stimulation in the company of others, or talkativeness) might be relevant for any business activity. In addition, attachment anxiety has been shown to be most responsible for the variability in becoming an entrepreneur, with secure attachment associated positively with the tendency to become an entrepreneur [35].

Importantly, personality traits are generally stable over the adult years [40, 41]. While most studies found increases in Openness to Experience in adolescence [49–52], few other studies reported negative trends [53, 54]. The findings for Agreeableness, Extraversion, Neuroticism, and Consciousness in adolescence are even less consistent [41]. In any event, most authors believe that personality changes occur primarily in young adulthood [55], and that the changes that occur after age 30 are quite modest [56].

Therefore, we hypothesize that:

*H2*: *Entrepreneurial tendency scores will associate with personality traits*.
*H2a*: *Entrepreneurial tendency scores will positively associate with Openness to Experience*, *Conscientiousness*, *and Extraversion*.
*H2b*: *Entrepreneurial tendency scores will negatively associate with Neuroticism and Agreeableness*.

Since age leads to an increase in human, financial, and social capital, these resources can offset the negative effect of age on entrepreneurial tendencies, especially in older people whose entrepreneurial potential is high. Since entrepreneurial activity increases with age for people who prefer to be self-employed and decreases with age for people who prefer to be hired by others [57], it is possible that in participants with high entrepreneurial tendency scores, the effects of personality traits would be even larger, and the contribution of age would be smaller. In addition, as personality traits (e.g., the Big Five) account for a large part of the variance in entrepreneurial intention [42, 43], we expect that traits that associate with entrepreneurship will continue to predict entrepreneurial intentions as people become older.

Therefore, in participants with lower entrepreneurial potential, as measured by their entrepreneurial tendency scores or by the fact that they are not entrepreneurs, the negative association between age and entrepreneurial tendency scores will be more pronounced than in participants with higher entrepreneurial potential. Hence, we hypothesize the following:

*H3a*: *The correlation between age and entrepreneurial tendency scores will be significantly larger in a sub-sample of participants whose entrepreneurial tendency is below the median tendency score relative to a sub-sample of participants whose entrepreneurial tendency is higher than the median*.
*H3b*: *The correlation between age and entrepreneurial tendency scores will be significantly larger in a sub-sample of non-entrepreneurs relative to a sub-sample of entrepreneurs*.

## Materials and methods

### Participants

The sample included 963 Hebrew-speaking adults (534 female, mean age = 45.7, SD = 15.9), 200 of them part time or full-time entrepreneurs, and 742 participants who were not entrepreneurs. The survey was conducted between 2016 and 2018 among 321 BA and MBA students (aged 19–53) in Business Administration at the Ono Academic College in Israel, and their 642 parents (aged 38–81). Students received a 5-point bonus in one of their courses if they and their non-student parents completed the survey. A research assistant contacted the parents directly in order to send them the survey and collect it after completion. The response rate of the parents was very high (over 95%), ruling out a potential bias due to different response rate between students and their parents. Table 1 shows the demographic characteristics of the sample. A minimal data set is included in the supporting information section (S1 File).

**Table 1:** Sample characteristics.

Variable	Average/Share	SD	Median
Age*	45.73	15.94	50.00
Female gender	55.45%	-	-
Religious	46.31%	-	-
Jewish	93.46%	-	-
Born in Israel	78.92%	-	-
Years of education**	13.22	3.42	13.00
Net household income***	13,136	10,382	8,500
Father entrepreneur	28.76%	-	-
Employee	65.32%	-	-
Self-employed	15.78%	-	-
Entrepreneur	20.77%	-	-
Took a course on entrepreneurship	8.62%	-	-

*Similar to the average age of the Israeli population over 19, which was 45.4 in 2018.

** Similar to the average education in the general Israeli population, which was 13.40 in 2011 for adults aged 25–64 (in the current sample, individuals in the age range of 25–64 had an average of 13.29 years of education).

*** Compare to 15,751 NIS in the general Israeli population. The difference is due to over-representation of students with very low income (33% of participants reported an income at the lower 20% of the Israeli general population). When correcting for this over-representation, the average net household income was very close to the average general Israeli population.

The institutional review board and ethics committee of the Ono Academic College approved the study. Informed verbal consent was obtained from each participant prior to participation.

We used the nQuery software (https://www.statsols.com/nquery) to determine the strength of using sub-samples. The minimum size of a sub-sample was calculated based on the mean and standard deviation of the entrepreneurial tendency score (mean = 204.6, SD = 27.9, maximum score 325). For a 9-point interval (a third of a standard deviation), the sub-sample should include at least 78 participants; for a 6-point interval, the sub-sample should include at least 172 participants; and for a 4-point interval, the sub-sample should include at least 384 participants (two-sided interval test, 95% confidence level).

### Materials

We used existing questionnaires to examine entrepreneurship tendency, personality traits (Big Five), attachment orientations, and demographic factors.

#### Entrepreneurship tendency scale

We used an updated version of [58] self-report scale. The scale consists of 65 items that assess four aspects of entrepreneurial personality: Entrepreneurial Awareness/Proactivity (e.g., "I am quick to spot ways of making money"); Entrepreneurial Creativity ("Even if I know how to do something, I would always try to do it in a different way"); Opportunism or Motivation ("When I see a business opportunity I jump on it without giving it much thought"); and Vision ("I am destined to make a difference in the world"). Total entrepreneurial potential score was calculated by adding scores of all individual items.

Respondents were instructed to rate each statement on a 5-point Likert scale that ranged from completely disagree (1) to completely agree (5). Cronbach’s α for the total scores was 0.90.

**Fig 1:**
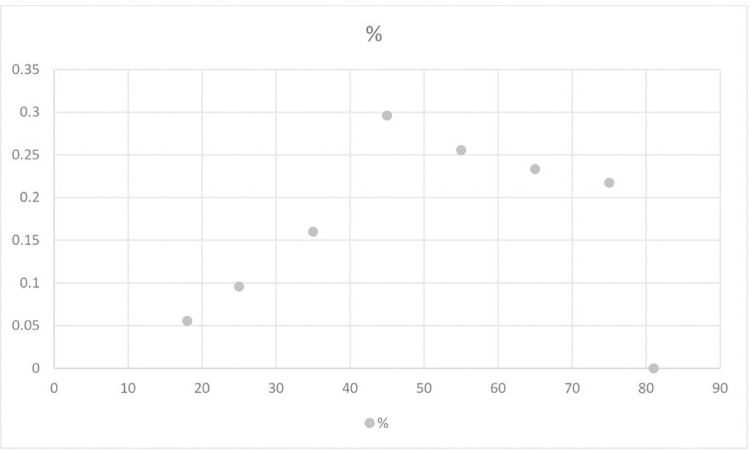
Distribution of actual entrepreneurship by age. The dots represent the percentage of actual entrepreneurs in each age group. Participants were asked if they were entrepreneurs at the time of the study. Older individuals might have been entrepreneurs prior to the study. Correcting for this possible bias would strengthen the inverted U-shape relationship between age and actual entrepreneurship.

The total entrepreneurial tendency score differed significantly between entrepreneurs and non-entrepreneurs (entrepreneurs average score = 219.72, non-entrepreneurs average score = 200.64, t = 9.8, p < 0.05). Furthermore, the relationship between actual entrepreneurship and age resembled an inverted U-shape with a maximum probability around age 45 (see Fig 1), as found in previous studies [30, 57, 59].

#### Big Five personality scale

We used the 44-item Big Five Inventory questionnaire [60, 61]. Eight items assessed Extraversion (E; e.g., "I see myself as someone who is talkative"); nine items assessed Agreeableness (A; e.g., "I see myself as someone who tends to find fault with others"); nine items assessed Conscientiousness (C; e.g., "I see myself as someone who does a thorough job"); eight items assessed Neuroticism (N; e.g., "I see myself as someone who is depressed, blue"); and ten items assessed Openness to Experience (O; e.g., "I see myself as someone who is original, comes up with new ideas"). Respondents were instructed to rate each statement on a 5-point Likert scale that ranged from completely disagree (1) to completely agree (5). Cronbach’s α’s were 0.75 for Extraversion, 0.73 for Agreeableness, 0.76 for Conscientiousness, 0.75 for Neuroticism, and 0.79 for Openness to Experience.

#### Entrepreneurial personality profile

[62] suggested that there is an entrepreneurial personality profile that can be calculated from the Big Five scores. Accordingly, we calculated the accumulated square distances of the scores of Openness to Experience, Conscientiousness, and Extraversion from the maximum score, as well as the square distances of the scores of Agreeableness and Neuroticism from the minimum score.

#### Attachment scale

We used the Experiences in Close Relationships scale [63]. This scale consists of 36 items, 18 items assessing avoidance (e.g., "I prefer not to show other people how I feel deep down"), and 18 items assessing anxiety (e.g., "I worry about being abandoned"). Respondents were instructed to rate each statement on a 5-point Likert scale that ranged from completely disagree (1) to completely agree (5). Cronbach’s α’s were 0.79 for avoidance and 0.88 for anxiety.

#### Demographic questionnaire

Participants were asked to provide background information on variables that are known to influence entrepreneurial tendency. These measures were used as control variables. Income and being an Arab minority or being an immigrant were included since necessity in general, and facing some kind of discriminatory hiring practices in particular, may affect the tendency to become an entrepreneur [4, 29]. Religion and degree of religiosity were included since different religious institutions have a different impact on the tendency to become an entrepreneur [64]. Parental entrepreneurial activity was recorded because parental practices affect ones’ tendencies to become an entrepreneur, such that having a parent who is an entrepreneur increases the probability that a child ends up as an entrepreneur by 30 to 200 percent [65–67]. Education was included as indication of human capital that may assist in the accumulation of knowledge, leading to the development of skills useful to entrepreneurs [68]. Gender was included since the literature documents a persistent entrepreneurial gender gap [69].

### Procedure

Students were invited to participate in a study on cultural influence on entrepreneurship. They were requested to ask their parents whether they agreed that an interviewer would contact them directly, explain the aims of the study, and leave the questionnaire with them to complete on their own. Parents filled the questionnaires at home and later returned them directly to the investigators. All participants were instructed to work through the packet of questionnaires at their own pace but in the same order of presentation. After completing the questionnaires, participants were debriefed and thanked for their participation.

### Statistical analyses

We used a logarithmic transformation on the raw data. Using natural logarithms is not supposed to change the direction of the conclusions but only to facilitate the analyses. Transformation into natural logarithms allows the coefficient to be expressed as percent change.

## Results

**Fig 2:**
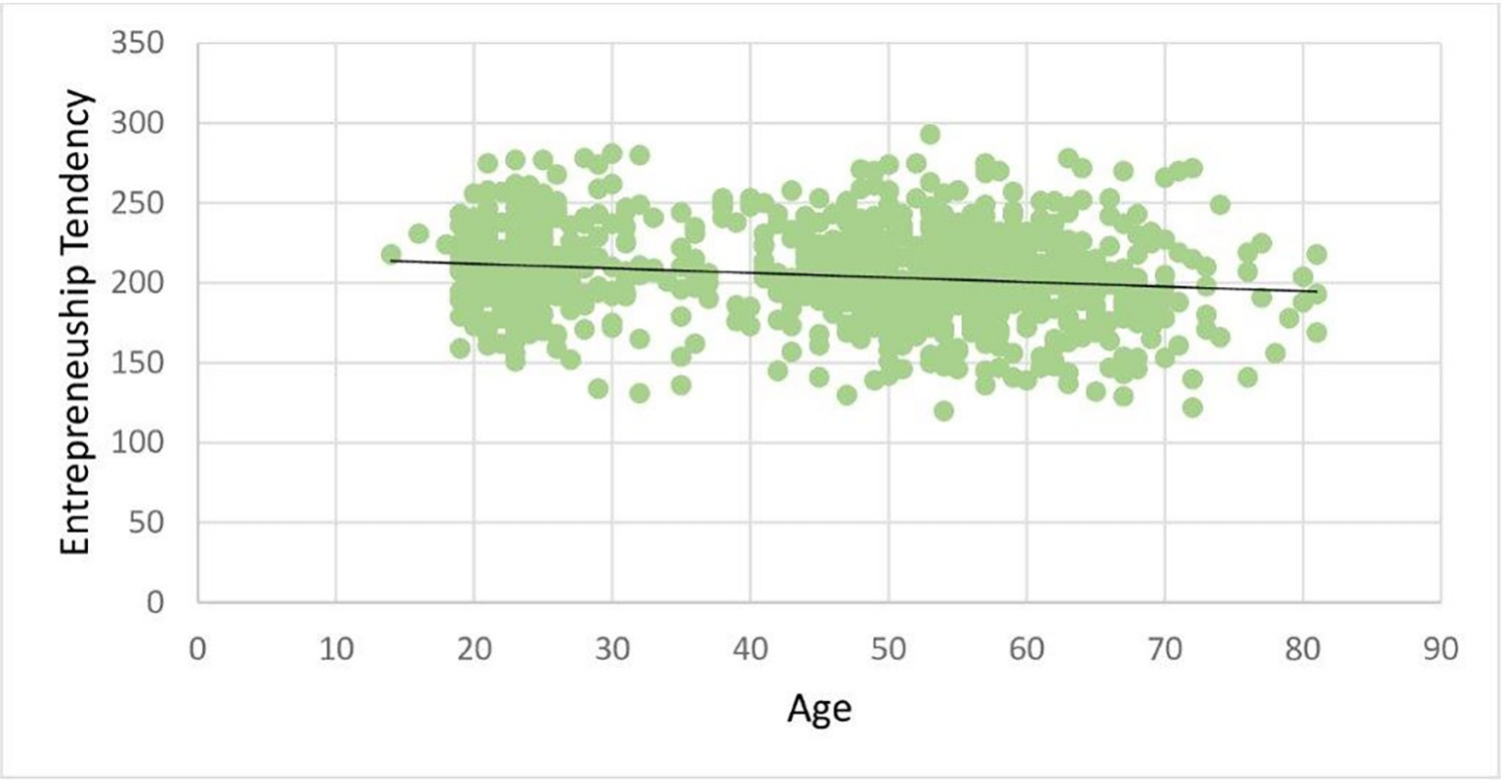
Distribution of the entrepreneurship tendency score by age.

Most variables were continuous variables. Binary control variables (e.g., gender) were treated as dummy variables. Table 2 presents total scores on the entrepreneurship scale by age groups, and Fig 2 shows the complete distribution of these scores. As the contrast between Figs 1 and 2 suggests, the relationship between age and actual entrepreneurship resembled an inverted U-shape, whereas the relationship between age and entrepreneurial tendency is almost a perfect flat line with a very limited slope.

**Table 2:** Means (and standard deviations) of the entrepreneurship tendency score, by age groups.

Age range	Number of participants	Number of entrepreneurs	Mean age	SD age	Mean entrepreneurship tendency score	SD entrepreneurship tendency score
18–20	36	2	19.25	0.00	213	16.26
21–30	230	22	24.15	2.26	210	26.75
31–40	50	8	34.60	3.09	210	16.11
41–50	179	53	46.98	2.34	206	26.37
51–60	305	78	55.16	2.55	202	25.20
61–70	137	32	64.77	2.52	198	33.72
71–81	26	5	75.15	1.92	194	38.81

### Age and entrepreneurial tendency in an uncontrolled model

In support of Hypothesis 1a, an uncontrolled OLS estimation revealed a significant negative correlation between age and the entrepreneurship tendency score. A linear regression model that predicted the entrepreneurship tendency score showed that age explained only 2.4% of the variance in scores (coefficient -0.055, Table 3, A-1).

**Table 3:** Estimation of equations explaining the entrepreneurship tendency score*.

Variable	Coefficients of version	Coefficients of version	Coefficients of version	Coefficients of version
	A-1	A-2	A-3	A-4
Estimation method	OLS	OLS	OLS	OLS
Constant	5.516+++	4.780+++	2.953++	7.019+++
	(133.883)	(42.415)	(2.087)	(52.332)
Age*	-0.055+++	-0.056+++	-0.569	-0.054+++
	(-5.002)	(-6.556)	(-1.437)	(-5.234)
Age root*	-	-	1.940	-
			(1.295)	
Gender	-	-0.028+++	-0.028+++	-0.023+++
		(-4.131)	(-4.228)	(-2.790)
Religiosity	-	0.014++	0.014++	0.020++
		(2.200)	(2.169)	(2.557)
Father entrepreneur	-	0.014++	0.015++	0.019++
		(2.011)	(2.081)	(2.158)
Employee	-	0.018++	0.014	0.022++
		(2.145)	(1.620)	(2.133)
Self-employed	-	0.035+++	0.032++	0.039++
		(2.736)	(2.441)	(2.495)
Part-time entrepreneur	-	0.034+++	0.033+++	0.047+++
		(2.704)	(2.624)	(3.046)
Full-time entrepreneur	-	0.045+++	0.045+++	0.052+++
		(3.940)	(3.888)	(3.700)
Taking a course on entrepreneurship	-	0.036+++	0.035+++	0.044+++
		(3.178)	(3.079)	(3.147)
Extraversion*	-	0.111+++	0.111+++	0.111+++
		(6.121)	(6.131)	(6.131)
Agreeableness*	-	-0.098+++	-0.099+++	-
		(-4.426)	(-4.475)	
Conscientiousness *	-	-0.087+++	-0.086+++	-
		(-3.931)	(-3.913)	
Neuroticism *	-	-0.063+++	-0.063+++	-
		(-4.995)	(-5.018)	
Openness to Experience	-	0.344+++	0.343+++	-
		(21.271)	(21.262)	
Insecure avoidant attachment*	-	0.015	0.015	0.233+++
		(1.063)	(1.063)	(11.408)
Insecure anxiety attachment*	-	-0.021++	-0.021++	-0.044+++
		(-1.970)	(-1.970)	(-2.752)
*R* ^2^	0.025	0.525	0.525	-0.023++
				(-2.033)
${\bar{R}}^{2}$	0.024	0.517	0.517	0.269
S.E.	0.137	0.097	0.097	0.260

* The equations are in log-linear form. Therefore, these variables are expressed in natural logarithms.

** The values in brackets are t statistics.

+ Significant at the 10 percent level.

++ Significant at the 5 percent level.

+++ Significant at the 1 percent level.

We conducted two further analyses to examine the correlation between age and the total entrepreneurship tendency score. First, we entered age and the square root of age to examine whether age effects are attenuated over the years, and alternatively we examined age and age square scores to examine possible acceleration of age effects. No significant effects emerged for these analyses. Second, we examined sub-samples of different ages to identify the point at which the correlation between age and the total entrepreneurship tendency score becomes non-significant. There was a significant negative correlation between age and entrepreneurship tendency scores in the sub-sample of 459 participants under age 50 (Table 4, B-2). In contrast, no significant association between age and entrepreneurship tendency scores was found within the sub-sample of 504 participants older than 50 (Table 4, B-1), or within the sub-sample of 163 participants older than 60.

**Table 4:** Estimation of equations explaining entrepreneurship tendency scores in selected sub-samples*.

Variable	Coefficients of version	Coefficients of version	Coefficients of version	Coefficients of version	Coefficients of version	Coefficients of version
	B-1	B-2	B-3	B-4	B-5	B-6
Estimation method	OLS	OLS	OLS	OLS	OLS	OLS
Sub-sample	Age 50+	Age up to 50	Below Median	Above Median	Entrepreneurs	Non -Entrepreneurs
			Entrepreneur ship	Entrepreneur ship		
			Tendency Scale	Tendency Scale		
Constant	3.961+++	5.066+++	5.283+++	4.490+++	4.363+++	4.909+++
	(9.919)	(32.625)	(51.272)	(27.360)	(21.176)	(45.157)
Age*	-0.065	-0.053+++	-0.016++	-0.049+++	-	-0.080+++
						(-5.765)
	(-1.009)	(-2.669)	(-2.038)	(-4.349)		
Gender	0.126+	-0.036+++	-0.015++	-0.010	-	-0.044+++
						(-3.521)
	(1.653)	(-3.314)	(-2.390)	(-1.220)		
Religiosity	-0.009	0.020++	-	0.013+	-	-
	(-0.693)	(2.210)		(1.694)		
Father entrepreneur	0.011	0.009	-	0.012	0.028++	-
				(1.214)	(2.038)	
	(0.734)	(0.942)				
Mother entrepreneur	-	-	-	-	0.040++	-
					(2.127)	
Employee	0.014	0.029++	0.020++	0.004	-	0.026++
						(2.176)
	(0.752)	(2.049)	(2.299)	(0.451)		
Self-employed	0.037	0.050+	0.027++	0.003	-	0.069+++
						(2.881)
	(1.626)	(1.943)	(2.411)	(0.175)		
Part-time entrepreneur	0.037	0.038++	0.022++	-0.032	-	-
	(1.575)	(2.121)	(2.298)	(-1.401)		
Full-time entrepreneur	0.057++	0.018	0.021++	0.054+++	-	-
	(2.974)	(0.823)	(2.169)	(3.094)		
Taking a course on entrepreneurship	0.033	0.036+++	0.021++	0.021	-	0.041+++
						(2.755)
	(1.228)	(2.660)	(2.228)	(1.218)		
Extraversion*	0.181+++	0.055++	0.059+++	0.075+++	0.090+++	0.099+++
	(4.227)	(2.380)	(3.309)	(3.418)	(3.100)	(3.900)
Agreeableness*	-0.013	-0.080+++	-0.076+++	-0.050++	-	-0.092+++
						(-3.200)
	(-0.239)	(-2.748)	(-3.235)	(-1.978)		
Conscientiousness *	-0.033	-0.117+++	-0.044++	-0.020	-0.103++	-0.093+++
	(-0.610)	(-4.038)	(-1.948)	(-0.757)	(-2.409)	(-3.180)
Neuroticism *	-0.027	-0.073+++	-0.048+++	-0.017	-0.079+++	-0.071+++
	(-0.953)	(-4.078)	(-4.215)	(-0.965)	(-3.331)	(-4.530)
Openness to Experience	0.288+++	0.374+++	0.194+++	0.220+++	0.363+++	0.344+++
	(8.548)	(15.886)	(9.173)	(12.014)	(10.745)	(15.270)
Insecure avoidant attachment*	0.025	-0.009	-0.023+	0.052+++	-	-
	(0.808)	(-0.426)	(-1.658)	(3.247)		
Insecure anxiety attachment*	0.025(1.139)	-0.035++	-0.018+	-0.006	-	-
		(-2.225)	(-1.812)	(-0.468)		
*R* ^2^	0.517	0.055++	0.321	0.356	0.531	0.476
		(2.380)				
${\bar{R}}^{2}$	0.486	0.541	0.302	0.332	0.517	0.465
S.E.	0.105	0.087	0.066	0.082	0.090	0.099

* The equations are in log-linear form. Therefore, these variables are expressed in natural logarithms.

** The values in brackets are t statistics.

+ Significant at the 10 percent level.

++ Significant at the 5 percent level.

+++ Significant at the 1 percent level.

We used the [70] method to estimate the strength of the likely bias for unobservable variables. The purpose of this method is to examine whether the results are driven by the absence of important variables that were not taken into account. The method is based on the comparison of two regression models, one with only one predictor and the other with all predictors. The ratio of the estimated coefficient of the single-predictor model to the difference between this coefficient and the coefficient of the full model provides an estimate of the effect of unobservable variables. The smaller the value of the coefficient difference is, the less the estimate is affected by the selection of control variables. The Altonji’s measure for age was 56. Therefore, to attribute the entire OLS estimate to selection effects, the effect of unobservable variables would have to be at least 56 times greater than the selection of the large set of control variables that were used, which in our view is very unlikely.

### Personality aspects and entrepreneurial tendency

As expected, and in support of Hypothesis 1b, personality aspects significantly predicted entrepreneurial tendency scores and accounted for most of the variance in the entrepreneurial tendency score.

Table 5 presents the distribution of the personality scores across age groups. In support of Hypothesis 2, personality traits contributed to the explanatory power of the model in general, with a large contribution of Extraversion and Openness to Experience in particular. The entire personality scale explained 35.8% of the variance in entrepreneurial tendency score out of 51.7% in total (Table 3, A-2).

**Table 5:** Mean scores on the personality trait questionnaire, by age groups.

Age Range	Extraversion	Agreeableness	Conscientiousness	Neuroticism	Openness to Experience
18–20	30.08	32.83	35.14	21.72	36.36
21–30	29.19	33.64	35.53	21.06	33.93
31–40	30.88	35.20	36.28	19.24	36.44
41–50	29.11	36.25	36.67	20.56	35.66
51–60	29.22	36.57	37.15	20.40	34.92
61–70	28.39	36.26	37.18	20.40	34.84
71–81	27.58	36.23	36.38	19.96	35.35
Total	29.15	35.55	36.54	20.56	34.96
Pearson correlation*	-0.030	0.209+++	0.121+++	-0.052+	0.033

*Based on version A-2 in Table 3 which is in log-linear form. Therefore, these variables are expressed in natural logarithms.

+ Significant at the 10 percent level.

++ Significant at the 5 percent level.

+++ Significant at the 1 percent level.

In support of Hypothesis 2a, Openness to Experience correlated positively with the entrepreneurship tendency score (coefficient 0.344, Table 3, A-2), and the same was true for Extraversion (coefficient 0.111, Table 3, A-2). Unlike the prediction of Hypothesis 2a, Conscientiousness correlated negatively with the entrepreneurship tendency score (coefficient 0.087, Table 3, A-2). In support of Hypothesis 2b, Neuroticism correlated negatively with the entrepreneurship tendency score (coefficient -0.063, Table 3, A-2), and the same was true for Agreeableness (coefficient -0.098, Table 3, A-2). In addition, insecure anxiety attachment correlated negatively with the entrepreneurship tendency score (coefficient -0.021, Table 3, A-2). Insecure avoidant attachment did not correlate significantly with the entrepreneurship tendency score.

Furthermore, we calculated the correlation between entrepreneurial tendency scores and the entrepreneurial personality profile. This analysis found a correlation of r = .384, p < .0001. However, using the entrepreneurial personality profile as a predictor accounted for only 26% of the variance in the entrepreneurial tendency score (Table 3, A-4) versus 51.7% of the variance in the model that included the separate traits (Table 3, A-2).

### Reassessing the contribution of age to the prediction of entrepreneurial tendency scores after taking personality aspects into account

Once all personality variables were taken into account, age explained only 1.3% of the variance in entrepreneurial tendency scores (Table 3, A-2 and A-3).

In addition, we looked at the interactions between age and each of the five personality traits, as well as at the interaction between age and the attachment orientations. No interactions were significant in predicting the entrepreneurial tendency scores. We then divided the sample according to the median score of each personality trait, and ran the models separately for the below-median and the above-median groups. There was no difference in the confidence levels of the coefficients of age in any of the below-median and above-median comparisons. These results suggest that no single trait is responsible for a change in the association of age and entrepreneurial tendency.

### Reassessing the contribution of age to the prediction of entrepreneurial tendency scores in participants with high entrepreneurial tendency

To examine Hypothesis 3a, we divided the sample into two sub-samples based on the median score for the entrepreneurship tendency score. Within the above-median sub-sample, age had a very small effect on the entrepreneurship tendency, with a coefficient of -0.016 (Table 4, B-3). Within the below-median sub-sample, the effect was significant, with a coefficient of -0.053 (Table 4, B-4).

To examine Hypothesis 3b, we looked for age associations within the sub-sample of 200 entrepreneurs versus a sub-sample of 763 non-entrepreneurs. Age was not a significant predictor of the total entrepreneurial tendency score within the sub-sample of entrepreneurs (Table 4, B-5), but it remained significant within the sub-sample of non-entrepreneurs, with a coefficient of -0.080 (Table 4, B-6).

### Control variables

In order to examine the robustness of the results, we included numerous control variables (Table 3, A-2). Being an employee versus being unemployed or retired correlated positively with the entrepreneurship tendency score. Being self-employed correlated positively with the entrepreneurship tendency score. Being a full-time entrepreneur, as well as a part time entrepreneur, correlated positively with the entrepreneurship tendency score. Both income and education (as well as their interaction with age) did not correlate significantly with entrepreneurship tendency. Being female correlated negatively with the entrepreneurship tendency score, but this correlation was not significant for the sub-sample of entrepreneurs (Table 4, B-6). The interaction of gender and age was not significant. Having a father (but not a mother) who was an entrepreneur was positively correlated with the entrepreneurship tendency score. Finally, being religious correlated positively with the entrepreneurship tendency score. Taking a course on entrepreneurship correlated positively with the entrepreneurship tendency score. However, none of these control variables were significant in the sub-sample of entrepreneurs (Table 4, B-6). Additional demographic variables, such as having a spouse or children, or coming from a country with a high level of entrepreneurship, were not significant.

## Discussion

Entrepreneurship clearly declines after the age of 50, but the cause of this decline is debated. Some theories have focused on external factors, especially social norms and age discrimination [28], whereas other theories have focused on the decrease in motivation for entrepreneurship [e.g., the OCT model, 8]. In the current study, we directly examine whether low entrepreneurial activity in old age is due to a decrease in the entrepreneurial tendency (with motivation as a significant component of tendency) and whether personality aspects continue to associate with entrepreneurial tendencies over time.

The results show that age has only a limited correlation with the tendency to become an entrepreneur. More importantly, the effect of age is seen primarily for participants with low entrepreneurship tendency and for those under age 50. These findings do not fit the predictions of the original OCT model that age would be negatively correlated with the entrepreneurship tendency, especially in the oldest individuals [8]. Thus, the decline in actual entrepreneurship is most likely not caused by a decrease in entrepreneurship tendency.

Furthermore, we suggest that personality traits that associate with high entrepreneurial tendencies continue to be prominent into old age. Therefore, as people grow older, those who have personality characteristics that associate with entrepreneurship (in particular high levels of Openness to Experience and Extraversion) become increasingly more differentiated from those who have personality characteristics that do not associate with entrepreneurship (high levels of Conscientiousness, Neuroticism, and Agreeableness). Indeed, in our sample age predicted only a small variance in entrepreneurship tendency scores in participants with high level of entrepreneurship tendency and none of the variance in participants who were actual entrepreneurs, but it correlated significantly with entrepreneurship tendency scores in participants with low tendency scores.

Moreover, the findings emphasize the importance of personality traits in characterizing the entrepreneurship tendency, and particularly the significance of the Openness to Experience and Extraversion traits. The relative stability of personality traits with aging fits well with previous reports that show that the main changes in personality occur in adolescence rather than later in life [55, 56]. The fact that personality traits have such a high explanatory power for the entrepreneurial tendency provides additional support to our argument that age by itself is not an important predictor of the decline in the tendency to become an entrepreneur.

The results for Openness to Experience, Neuroticism, Extraversion, and Agreeableness are in line with the empirical literature about entrepreneurship and personality traits [42, 43]. However, the negative correlation between Conscientiousness and the entrepreneurship tendency appears to contrast previous findings [42, 43]. It is important to point out, though, that [42] did not examine the correlation between personality traits and the entrepreneurship tendency within the general population, but instead they compared entrepreneurs to managers. It is possible that the same negative correlation that we found for Conscientiousness would have been found in managers as well, even though they differ from entrepreneurs on this trait. In our dataset, the correlation between the entrepreneurship tendency and Conscientiousness was not significant in participants with a low entrepreneurship tendency, but we note that managers are not necessarily low on this tendency.

The results also indicate that the potential to become an entrepreneur is greater in people who are active in the working force than in individuals who are unemployed or in those who have already retired. Perhaps work encourages people to identify business opportunities in order to aim for achievement, either through entrepreneurship or through intrapreneurship (i.e., a process of entrepreneurship inside an existing business unit). Indeed, the entrepreneurship tendency scale is supposed to capture both entrepreneurship and intrapreneurship. If this is the case, older adults might not exercise their entrepreneurship potential because once they retire, they may find it more difficult to imagine themselves establishing a new business.

To sum up, our results may offer a novel explanation to the gap between the inverted U shape that characterizes age and actual entrepreneurship and the almost perfect flat line that characterizes age and entrepreneurial tendency. We suggest that three forces underlie this pattern of results:

- Entrepreneurial tendency–this tendency is related primarily to personality traits and therefore remains stable into old age, especially for people with high entrepreneurial tendency.
- Internal barriers to entrepreneurial execution–these barriers decrease with age because human and social capital as well as financial means accumulate with age. However, it is possible that this positive force is weakened with age due to various factors such as the effects of retirement.
- External barriers to entrepreneurial execution–these barriers increase with age as discriminatory practices and social exclusion become more prominent.

The combined effect of the first two forces can explain the difference between people with low entrepreneurial tendency and people with high entrepreneurial tendency. If one has a low entrepreneurial tendency, there is nothing to balance the increasing negative effect of the Opportunity Cost of Time [OCT model, 8]. In contrast, if one has a high entrepreneurial tendency, one can benefit from the increased effect of experience, human, social, and financial capital that have been accumulated over the years. However, external barriers affect both types of people.

### Alternative explanations and limitations

We acknowledge that our research has some limitations. First, the analyses were based on a cross-sectional design and thus casual conclusions should be cautious. Moreover, this design raises the possibility that the negative correlation between age and the entrepreneurial tendency scores, although limited, might reflect the effects of one’s birth cohort. That is, there is always the possibility that younger generations are more inclined toward entrepreneurship. We believe that this is not a major concern, since the explanatory variables identified in the entrepreneurship literature exhibit considerable consistency across the lifespan and in various sub-samples. Had there been a birth cohort effect, it should have been more pronounced in earlier born cohorts, leading to greater decline of the entrepreneurship tendency with age. However, the negative correlation between age and the entrepreneurial tendency was significant for participants up to age 50 and not significant in older cohorts. Even though the possibility of significant birth cohort effects is unlikely, future research could benefit from a longitudinal study of changes in entrepreneurial tendencies.

As for the generalizability of the results, it is important to note that the younger part of the sample consisted of students in business programs who might express a higher entrepreneurship tendency than other people their age. In addition, we did not control for health problems that might decrease entrepreneurship tendencies, especially in older adults. Nevertheless, if the youngest participants in the current sample present the upper limit of the entrepreneurship tendency and the older participants present the lower limit of the entrepreneurship tendency, the effect of age in the general population after controlling for health problems should be smaller than documented here. Therefore, if indeed there is a bias it worked against our hypotheses.

Finally, there may be distinctions between innovative/technology ventures and traditional/low tech ventures. In this regard, the external barriers, the internal barriers, and the tendency of older entrepreneurs to establish these two types of ventures might be different. For example, under the OCT model, it is possible that the cost of time effect on the general tendency for entrepreneurship is less pronounced, but the effect is more salient depending on specific type of ventures (e.g., products versus services or innovative/technology versus traditional/low tech ventures). Our data cannot allow us to make such distinctions.

## Conclusions

This study examines whether one’s entrepreneurial tendency decreases with age, whether this decrease accelerates in the oldest population, and whether the tendency to engage in entrepreneurship is stable after taking into account psychological traits that affect entrepreneurship. The results challenge the OCT model and emphasize the importance of considering personality traits as significant predictors of entrepreneurship tendencies across the lifespan. Once personality traits are entered into the prediction models, age has a limited effect on people’s tendency to become entrepreneurs.

These results call policy makers to focus on the external societal barriers that prevent older adults from executing their entrepreneurial tendencies. Since it is more difficult for policy makers to change people’s entrepreneurial cognitive structure or dismantle motivational barriers than to remove external societal barriers, the findings hold promise that older adults will engage in more extensive entrepreneurial activity in the future.

## Supporting information

S1 File (XLSX)
